# The Impact of Coronavirus Information-Seeking Behavior on Dental Care Access: A Cross-Sectional Questionnaire-Based Study

**DOI:** 10.3390/ijerph182212050

**Published:** 2021-11-17

**Authors:** Silvia Gallegati, Luca Aquilanti, Valerio Temperini, Gloria Polinesi, Giorgio Rappelli

**Affiliations:** 1 Department of Management, Polytechnic University of Marche, Piazzale Martelli 8, 60121 Ancona, Italy; v.temperini@staff.univpm.it; 2 Department of Clinical Specialistic and Dental Sciences, Polytechnic University of Marche, Via Tronto 10, 60126 Ancona, Italy; l.aquilanti@pm.univpm.it (L.A.); g.rappelli@staff.univpm.it (G.R.); 3 Department of Economic and Social Sciences, Polytechnic University of Marche, Piazzale Martelli 8, 60121 Ancona, Italy; g.polinesi@staff.univpm.it; 4 Dentistry Clinic, National Institute of Health and Science of Aging, IRCCS INRCA, Via Tronto 10, 60126 Ancona, Italy

**Keywords:** COVID-19, mass media, dental care access, gender differences, survey, consumer perception

## Abstract

Health information-seeking behavior provides a variety of benefits, such as reducing knowledge gaps and educating individuals outside the medical office. This study aimed at evaluating if different sources used to gather information on COVID-19 could affect the willingness to undergo dental appointments. An anonymous survey was posted on social media. The 1003 respondents used several channels of communication, clearly distinguishing reliable from unreliable ones. Multiple logistic regression estimated the effect of different information channels on the probability of being strongly influenced by COVID-19 in accessing upcoming dental appointments. Newspapers were the most-used channel of information (61.2%), blogs and forums the least used (11.2%). Overall, the more an individual was informed, the higher was the risk of missing upcoming dental care appointments (OR 2.05, CI 1.45–2.90, *p* < 0.001). The two most reliable channels of communication were identified in journals/websites of medicine and healthcare professionals. Women proved to be more active in gathering information and relying on less secure but more personal channels, such as social media and friends and family, thus having an increased risk of being influenced by COVID-19 information regarding upcoming dental care appointments (OR 3.62, CI 0.85–15.52, *p* < 0.1 and OR 1.60, CI 1.00–2.58, *p* < 0.1, respectively). Social media should have a greater presence on the side of medical service providers to avoid distortions of information and fake news that ultimately cause fear among citizens and compromise their health. Healthcare professionals and institutions should adapt their communication channels based on the audience they want to address to optimize the education and information of the final users.

## 1. Introduction

The severe acute respiratory syndrome coronavirus-2 (SARS-CoV-2) pandemic has posed a challenge to healthcare systems across the world, and its rapidly evolving spread has become a global health crisis. In this scenario, Italy has been severely affected by COVID-19, registering more than 4.5 million cases since the beginning of the pandemic and reaching more than 130,000 deaths.

Soon after the declaration of the pandemic by the WHO, national governments started issuing advisories and regulations to their people with the aim of restricting the spread of SARS-CoV-2. These policies included several limitations, ranging from social isolation to travel and movement restrictions. Governments used print media, mass media, and the web to inform the community. The communities themselves used a variety of tools and media sources, predominantly web based, to learn more about COVID-19: people started looking for symptoms and precautionary measures to avoid contagion [1].

During a disaster or a public health emergency, information sources help people be more conscious and aware of the situation, learn precautionary measures, and reduce anxiety caused by the uncertainty of a newly emerged situation [2]. Health information-seeking behavior, meant as the “purposive acquisition of health information from selected information carriers” [3], can provide a variety of benefits, above all the potential to reduce knowledge gaps across social groups and educate individuals outside the doctors’ office [4]. At the same time, information sources can create new problems, especially if not properly accessed by the people. Scholars believe that new media, especially social media, have great potentials to support information searching and decision making on self-care and health-related issues [5].

The proliferation of new media active in healthcare poses several problems and challenges [6]. First of all, the quality of health-related information present on social media is far from perfect, with contents being misleading, inconsistent, or not trustworthy [7]. Secondly, the excessive internet use recorded may have led to both cyberchondria and information overload [8,9]. An overexposure to information can increase the initial rates of post-traumatic stress disorder symptoms and can positively be related to forming risk perceptions. Nevertheless, Farooq et al. stated that, during the SARS-CoV-2 pandemic, cyberchondria and information overload contributed to the adoption of recommended health behavior, suggesting that strategies aimed at motivating citizens should focus both on stressing the gravity of the situation and on enhancing the quality of information through the reduction of overloading information [10].

Previous studies investigated how information sources have different impacts on psychological well-being and coping behavior during COVID-19, highlighting different results. Chao et al. have proved that information seeking on social media is generally associated with adverse psychological outcomes, while this negative relation does not apply to traditional mass media, such as television and the press [2]. Additionally, health information-seeking behavior has varied during the pandemic. COVID-19 has reached great public attention as a result of information-seeking behavior, and a considerable amount of information about the virus, its outbreak, and spread has been published and searched [11].

Although in Italy, dental offices practice was never formally stopped by specific policies regulating the spread of coronavirus, doctors suspended routine dental treatments, limiting their practice to just urgent and emergency dental care. In addition, the anxiety and fear generated by the spread of SARS-CoV-2 affected the willingness of the population to undergo dental treatment, further restricting access to dental care [12]. Especially during the very first phases of the pandemic, dental practice and dental professionals were considered to be subjected to a high risk of becoming infected with SARS-CoV-2. However, recent studies showed that the risk of SARS-CoV-2 transmission in dental settings is low, as the adherence to national infection control guidelines is high among clinicians [13,14,15].

Several studies have investigated the levels of anxiety and risk perceptions around the world during the COVID-19 pandemic so far, but, to the best of our knowledge, little attention has been focused on how different channels of communication could affect the availability to access dental care in the future [16]. With this background in mind, the aim of this study was to assess whether access to different communication tools (online, offline, social media, etc.) by the Italian population, with the purpose of seeking coronavirus-related information, as well as the level of trust shown by the population toward these sources of information, could somehow influence the availability to undergo dental treatment.

## 2. Materials and Methods

Questionnaires are useful tools for investigating the perspectives and attitudes of a population. For this study, an online survey was conducted using the free-access platform powered by Google (https://forms.gle/sAP8E5D3pWgs9pn46, accessed on 19 May 2020). Data collection occurred between 11 May and 18 May 2020, and a total of 1003 responses were obtained. The questionnaire, in the Italian language, was composed of 43 questions, divided into 3 sections (Table S1). The first segment was designed to obtain socio-demographic information (age, gender, level of education, employment) and to survey the main financial consequences (i.e., family income) caused by the SARS-CoV-2 pandemic and the consequent modifications to the lifestyle it brought along. The following sections were aimed at understanding the relationship between information sources and future availability to access oral care after the pandemic. Respondents were asked about a variety of channels of information used to obtain information on COVID-19 and, for each channel, they were asked to assign a value of reliability ranging from 1 (not reliable) to 5 (very reliable). The questions were aimed at comprehending how trust in different communication means could influence the willingness of Italian individuals to undergo dental treatment.

Initially, the questionnaire was revised by experts (expert colleagues in statistics, economics, and medicine), and a pilot study was conducted with a group of 20 people, heterogeneous for demographic traits, education, and employment in order to assess the understandability of the questionnaire. On the basis of the respondents’ suggestions and reactions, some questions were adjusted to make the questionnaire more understandable and clearer. The study was based on the questionnaire previously published and analyzed elsewhere [12]. In particular, the purpose of the analysis was to assess how much means of communication used to receive information and news on the SARS-CoV-2 pandemic were perceived as reliable by the population and whether this perceived reliability would affect the willingness of the Italian people to access upcoming dental care appointments.

For this study, it was deemed appropriate to use a non-probability sampling technique called snowball sampling that has the advantage of expanding the sample size, reducing at the same time cost and time of research [17]. Moreover, snowball sampling allowed the recruitment of participants that were not easily accessible to the researchers because of the lockdown imposed by the pandemic [18,19,20]. The self-administrated standard anonymous questionnaire, composed of 43 questions, was posted on social media belonging to the Facebook Group (Facebook, Instagram, and WhatsApp), on the personal accounts of two members of the research group. Using the snowball approach, all the respondents were asked to share the link with other people [21,22].

Several research studies have demonstrated that Facebook is an effective tool for data collection: snowball sampling adopted on Facebook manages to provide higher response rates than traditional snowball sampling, because of the strong personal links that exist on the social network between the researcher and the respondents [17,23]. Social media platforms belonging to the Facebook group were chosen as preferred social media for the purposes of the research because of their increasing relevance as social networks, as communication tools for the users, and also for the significant amount of time that users spend on these platforms [23].

This study was conducted in full accordance with national and international regulations and the Declaration of Helsinki. All the respondents were fully informed about the aims of the study and were required to accept informed consent about the data sharing and privacy policy before participating in the survey, in compliance with Italian Legislative Decree 196/2003 and UE GDPR 679/2016. The anonymous nature of the questionnaire did not allow the identification of any personal data.

### Statistical Analysis

Descriptive analysis was initially performed to evaluate the main characteristics of the participants in the study. Absolute and percentage frequencies were calculated to summarize the characteristics collected. Descriptive statistics were performed, and the dependent variable influence of COVID-19 information-seeking behavior on accessing future dental appointments (from now on, just “influence” for brevity) was introduced in a multiple logistic regression to estimate the effect of different sources of information on the probability of being influenced by COVID-19 in accessing future dental appointments. This binary variable was defined as “no” or “yes”, on the basis of the influence of COVID-19 on future dental appointments. In particular, the variable was defined as “no” if the corresponding value of the question n. 38 (Table S1) was minor, or equal to 3, while it was defined as “yes” if it was major, i.e., more than 3.

Statistical analyses were performed with Stata statistical software, and the outcomes were considered both at the level of the whole sample and at the level of two subgroups divided by gender (male and female), in order to investigate possible gender differences in the willingness to undergo future dental treatment, also considering the impact of the communication channels used by the respondents to gather information about COVID-19.

## 3. Results

**Figure 1:**
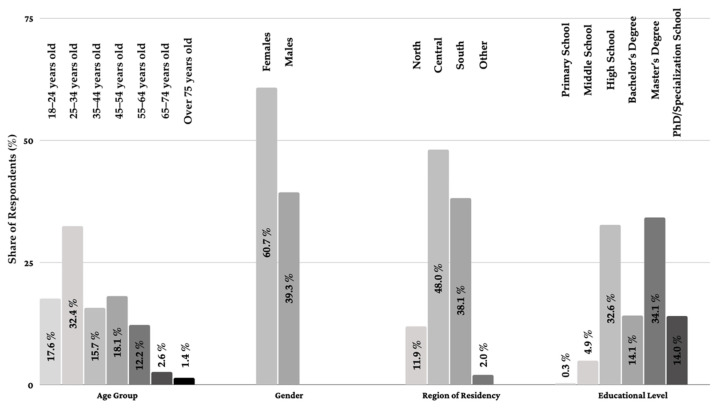
Demographic information of the respondents to the questionnaire (*n* = 1003).

Overall, out of the total 1003 respondents, 60.7% were female and 39.3% male. The largest share of respondents belonged to the age group 25–34 (32.4%), followed by the age groups 45–54 (18.1%), 18–24 (17.6%), 33–44 (15.7%), 55–64 (12.2%), 65–74 (2.6%) and over 75 (1.4%). Figure 1 describes the characteristics of participants.

**Figure 2:**
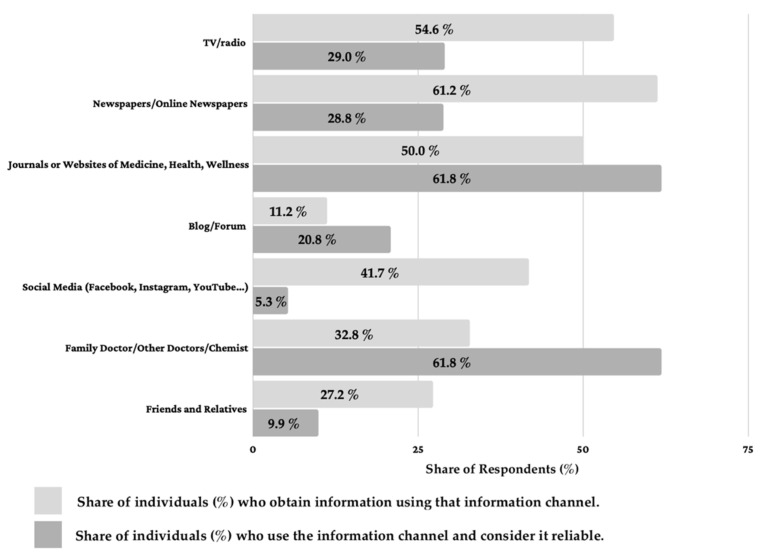
Information channels used by the respondents to the questionnaire (*n* = 854). Light grey refers to the % of individuals who obtain information using that information channel. Dark grey refers to the % of who use the information channel and consider it reliable.

After dropping missing values of the dependent variable influence, statistics were carried out on 854 individuals, with the purpose of analyzing how different communication means were used to gather information concerning COVID-19. Figure 2 shows results for the seven information channels considered: television, newspapers, journals or websites of health and medicine, blog/forum, social media (e.g., Facebook, YouTube, and Instagram), friends and relatives, chemists, and family doctors.

It was revealed that respondents collect information from several means of communication, but they clearly distinguish reliable channels (assigned value of 4 and/or 5) from unreliable ones (assigned value of 1, 2, and 3), in terms of the quality of the information provided. The most used source of information was newspapers/online newspapers, followed by TV/radio, while the least used sources were blogs and forums.

Focusing on the number of individuals who believe the information to be true, journals or websites of medicine, health, wellness, and family doctor/other doctors/chemists were considered the most reliable channels, followed by TV and newspapers or online newspapers. Few individuals seek information from blogs and forums, while the information provided by social media and friends, despite being accessed by many, was generally not reliable for most of the respondents.

The interaction terms used as predictors in the logistic regression model took into account the following information channels: TV/radio, newspapers/online newspapers, journals or websites of medicine, health, and wellness, blog/forum, social media (e.g., Facebook, YouTube, and Instagram), friends/relatives and family doctor/other doctors/chemist. The interaction terms were dichotomous variables, assigned a value of 0 if the respondent did not gather information from that specific channel, 1 if otherwise. 

The associated variable reliability represents the trust of respondents with reference to the corresponding information channel. In question n.16 (Table S1), respondents were asked to assign a level of perceived reliability to each channel of communication investigated: the values were ranging from 1 (not at all) to 5 (extremely). During the analysis, the reliability variable was converted into a dichotomous one: if the respondents showed trust toward the investigated information channel (assigning values of 4 and/or 5), the value of the dichotomous variable reliability reached 1; otherwise, in case the respondents did not trust the channel of communication, the value reached 0.

Table 1 shows the association among information means and the influence of COVID-19 on upcoming dental appointments for the whole sample and for the two population subgroups of females and males. Odds ratio estimates have been computed also for combinations of variables (e.g., social media and reliability). The communication tools that were used by the respondents and, at the same time, were deemed to be reliable were considered (reliability = 1). The purpose was to understand to what extent using and trusting a communication channel could influence the willingness to undergo dental appointments.

Multiple logistic regression showed that the risk of being influenced by COVID-19 information as regards upcoming dental care appointments, relative to the risk of belonging to the “no influence” category, was 2.05 times higher (more than double) for people who spontaneously gathered further information to feel safer with concern to access to oral care, regardless of the sample dimension considered. In other words, the more an individual becomes informed about COVID-19, the higher is the risk that the upcoming dental care appointments will be affected. On the contrary, if the information gathered included all the necessary details on the sanitization procedures adopted in the dental office, then the risk of being influenced by COVID-19 information in relation to upcoming dentist appointments decreased, but this was only valid for males. Moreover, it was also found that individuals feeling informed and having a higher degree of education (bachelor, master or PhD) showed a risk of being influenced by COVID-19 information as regards upcoming dentist appointments that was 1.66 times higher for the whole sample and 2.42 for the male group (*p* < 0.05). Focusing on gender differences, results showed that women tended to perceive less secure channels such as social media and friends as reliable sources, differently from males, who preferred to gather information from the family doctor, hence preferring more institutional sources. For both subgroups, collecting information from journals and newspapers increased the risk of being influenced by COVID-19 information (compared with not being influenced) by 1.68 times (*p* < 0.01).

## 4. Discussion

The current study aimed at investigating the factors affecting consumers’ availability to undergo future dental appointments after the SARS-CoV-2 pandemic, focusing on users’ information-seeking behavior and attempting to assess differences over risk perception depending on the channel of information used. The presented results showed that communication tools affect the risk of being influenced by fear of contagion by COVID-19 as regards accessing dental care, but also that there are demographic and especially gender differences that influence the risk perception over future visits at the dentist.

In this study, a significant positive relationship was found between information about COVID-19 and the perceived risk of infection: this finding is consistent with previous studies on the Middle East respiratory syndrome (MERS) [24,25], demonstrating that MERS-related knowledge was significantly correlated with preventive behaviors. This may imply that individuals who are more informed about COVID-19 and its modalities of contagion are also more aware of the high infectious characteristics of the disease and hence less willing to be exposed to the risk of contagion through dental care.

From the analysis, it was also revealed that communication is a key point in determining patients’ availability to undergo dental care: individuals who seek health-related information today have access to many more sources than they had in the past. While knowledge can be acquired from several different means of communication, patients clearly understood that reliability was not equal for all. This study highlighted that health professionals such as family doctors and chemists were entrusted as the most reliable source of information about COVID-19, on par with journals or websites of medicine, health, wellness. The finding is in accordance with previous studies on COVID-19 and on H1N1 influenza [26,27].

Confirming our results, studies also show that, already in 2004, the internet was one of the main information sources, and it was already expected to play a key role in healthcare communication in the future [28]. In 2008, the internet was the second-most popular source for seeking health-related information, although its believability was found to be relatively low [29]. The aspect of believability is confirmed by this study, especially for what concerns social media, since only 5.34% of users looking for information on these channels consider the information gathered reliable. The evident discrepancy between the most credible and most accessible source of information (family doctor/other doctors are most credible with 61.83% but second-least accessible with 32.79%) should be covered to enhance COVID-19-related communication and minimize risks of negative influence over the population. In this sense, health professionals and family doctors, in particular, should enhance their presence on social media platforms, and play a more focal role in delivering information about COVID-19, and contact more widely and more often with their patients (i.e., using broadcast lists, newsletters, mailing lists, etc.) to convey secure and reliable information. The presence of dentists on social media should also be enhanced in light of the fact that the COVID-19 outbreak increased the use of social media communication because patients feel anxiety about infection through close contact [30].

The study also highlighted severe discrepancies between health information-seeking behavior and risk perceptions of men and women: in fact, not all groups sought health information equally. Females were more likely to engage in information seeking than males. Gender is a factor that discriminates e-health information quality perception, with males perceiving e-health information quality lower than females [31]. Our results also confirmed previous studies according to which women rather than men tended to gather more health-related information [32]. This may be due to the fact that women are more interested in health-related issues because of the care-taking role they generally have in families, and this makes them more aggressive in searching for this kind of information [33]. Women tended to search for information on a wider variety of information channels compared with men, becoming more involved in quality evaluation of e-health information than males but also trusting less secure channels such as personal networks (family, friends) and social media. Our results also confirmed those of the study by Bidmon and Terlutter (2015) according to which, in comparison with men, women reported a higher frequency of using health forums and blogs, and the internet was a more important source of information for them [34].

Nevertheless, social media appeared to be a prominent and emerging information channel. Parmar et al. highlighted that social media may offer critical opportunities for dentists to facilitate patient-dentist communication and that 44% of the patients make contact with their physicians through social media [35]. Especially for women, it would be appropriate to create official social media channels to convey information and raise operational efficiency to create patient-friendly services. Since women may be more easily convinced by health awareness information found online, they should be a primary target group for this kind of communication, and this should be interesting for government institutions (i.e., for health consciousness campaigns) but also for doctors wanting to promote their activity or reaching out to their patients. In this sense, social media use has the potential to improve health by spreading health information and may allow dentists to deliver the service and support outside the dental practice environment, increasing patients’ trust and the appreciation of the services received [36].

For men, who, in this study, demonstrated to rely on secure and official channels such as family doctors, teledentistry, started by the US military in 1994 to serve the US troops all around the world, can provide an innovative solution to continue dental practice during pandemics and beyond [37]. Today, teledentistry needs to be implemented into routine dental practice: if not fully replaced, it can at least implement the compromised dental system during the pandemic.

This study has certain limitations that provide opportunities for future work. As the snowball sampling technique is a non-probabilistic method of sampling, it may limit the representativeness of the sample because of self-selection bias, despite having been proven to be an efficient tool for data collection. Snowball sampling through social media may introduce sampling bias toward characteristics of those who have an active online presence [38]. This study, despite not being population based, still managed to reach almost all Italian regions with a wide range of demographic characteristics of the respondents. Future research extending the current study should recruit a more diverse sample to overcome these limitations and broaden the interpretation of results. Another limitation of this study may be that gender differences are likely to be dependent on the cultural background, so we cannot consider the results generalizable beyond the Italian population, even if comparable studies in other countries could confirm the generalizability of our findings. The data analyzed in this study refer to the early phases of the pandemic, in May 2020, when the levels of fear and anxiety were at very high levels [39]; the quality of data collected is very much time related, but it has also great importance because the research allowed the perception of patients to be analyzed in the early moments of the emergency and to draw implications in the physician–patient relationship that will be applicable in daily communication strategies and in case of future emergencies.

The purpose of this study was to understand the influence of seeking knowledge about COVID-19 on the Italian population with a specific reference to the willingness to undergo upcoming dental care appointments. Several factors, such as the variety of information channels used and some demographic variables such as gender were the focal points of the analysis. It was clearly evident that respondents collect information from a wide variety of means of communication, but they distinguish reliable channels from unreliable ones. In general, it was revealed that gathering information about COVID-19 increases the risk of being influenced over future dental appointments: this may be due to the fact that an informed individual is more conscious about the modalities of contagion by SARS-CoV-2 and perceives a higher level of risk, in line with the results by Motta Zanin et al. on overestimation of risk perception during COVID-19 [39]. From the current study, it also emerged that there are clear differences in the information sources used by men and women: men tend to rely on official sources such as health care professionals and family doctors, while women, who, on average, search for more information probably also because of their care-taking role in families [34], tend to rely on less secure sources such as personal links (family, friends) and social media.

This study revealed important implications for dental service providers and for other healthcare services professionals in general. In particular, the opportunity to develop an adequate presence on social channels was highlighted in order to educate and provide useful information to users [30,35,36], especially if the target audience is female, given that women are more likely to rely on online sources. The fact that the source of information is institutional or represented by a healthcare professional can provide greater credibility and reassure users to a greater extent; in fact, there is a certain awareness that misleading information and news mostly circulate on the web. In the case of male users, trust can be gained through direct doctor–user communication, and therefore, it becomes essential to develop the relationship between them.

## 5. Conclusions

In the event of a crisis such as that generated by the COVID-19 pandemic, social media can play an important role in serving as a source of information and in addressing people’s opinions: social media should hence have a greater presence on the side of medical service providers in order to avoid distortions of information and fake news that ultimately cause fear among citizens and compromise their health by avoiding, for example, preventive checks or unnecessarily postponing necessary interventions.

Taking into consideration these results, healthcare professionals and institutions should be able to adapt their communication channels based on the audience they want to address, with the purpose of conveying official and secure information on health-related issues such as COVID-19.

## Figures and Tables

**Table 1:** Odds ratio estimates of variables associated with influence of COVID-19 on upcoming dentist appointments (* *p*-value < 0.10, ** *p*-value < 0.05, *** *p*-value < 0.01, **** *p*-value < 0.001).

Independent Variables	All (95% CI) *n* = 854	Females (95% CI) *n* = 519	Males (95% CI) *n* = 335
Look for further info on COVID-19	1.44 (0.90–2.31)	1.81 (0.99–3.31) *	0.85 (0.38–1.90)
Feeling informed	0.70 (0.45–1.10)	0.66 (0.37–1.19)	0.83 (0.39–1.74)
Look for further info about oral care	2.05 (1.45–2.90) ****	2.22 (1.36–3.63) ***	1.91 (1.12–3.24) **
Information by dentist sanitization procedures	0.64 (0.46–0.88) ***	0.71 (0.46–1.10)	0.52 (0.31–0.88) **
Feeling informed + be graduated	1.66 (1.08–2.56) **	1.25 (0.70–2.23)	2.42 (1.21–4.85) **
TV/radio	0.77 (0.53–1.13)	0.76 (0.46–1.24)	0.79 (0.42–1.50)
Newspapers/online newspapers	1.68 (1.14–2.50) ***	1.70 (1.02–2.84) **	1.80 (0.95–3.40) *
Friends and relatives	1.25 (0.86–1.82)	1.60 (1.00–2.58) *	0.98 (0.50–1.91)
Social media (Facebook, Instagram, YouTube, etc.)	0.98 (0.69–1.40)	0.86 (0.54–1.35)	1.18 (0.65–2.14)
Blog/forum	0.74 (0.41–1.34)	0.69 (0.32–1.48)	0.74 (0.28–1.98)
Journals or websites of medicine, health, wellness	1.11 (0.70–1.75)	1.32 (0.73–2.39)	1.07 (0.49–2.35)
Family doctor/other doctors/chemist	1.28 (0.80–2.06)	1.03 (0.55–1.92)	1.93 (0.87–4.24)
Social Media + reliability	1.70 (0.60–4.84)	3.62 (0.85–15.52) *	0.90 (0.16–5.03)
TV/radio + reliability	0.90 (0.52–1.57)	1.05 (0.52–2.13)	0.77 (0.30–1.96)
Newspapers + reliability	1.11 (0.68–1.83)	0.88 (0.46–1.66)	1.65 (0.70–3.89)
Blogs + reliability	2.40 (0.76–7.61)	1.59 (0.26–9.61)	2.18 (0.40–11.80)
Journals/websites of medicine + reliability	1.27 (0.79–2.04)	1.14 (0.62–2.10)	1.36 (0.61–2.99)
Family doctor + reliability	0.76 (0.43–1.36)	1.09 (0.51–2.29)	0.43 (0.17–1.12) *
Friends + reliability	1.74 (0.68–4.49)	1.07 (0.31–3.63)	3.85 (0.73–20.41)

## Data Availability

The datasets generated and/or analyzed during the present study are available from the corresponding author on reasonable request.

## Supplementary Materials

The following are available online at https://www.mdpi.com/article/10.3390/ijerph182212050/s1, Table S1: This is the English version of the original questionnaire in the Italian language (https://forms.gle/sAP8E5D3pWgs9pn46, accessed on 19 May 2020).
