# Supplementary material for: The Impact of Coronavirus Information-Seeking Behavior on Dental Care Access: A Cross-Sectional Questionnaire-Based Study
Source: Int J Environ Res Public Health. 2021 Nov 17;18(22):12050. doi: 10.3390/ijerph182212050 (PMC8622317; doi:10.3390/ijerph182212050)
Supplement: Supplementary file 1 [file ijerph-18-12050-s001.zip › ijerph-1419959-supplementary.pdf]

Table S1. This is the English version of the original questionnaire in the Italian language (https://forms.gle/sAP8E5D3pWgs9pn46).

Question	Variables
**Q1. Select your age group**	18–24
	25–34
	35–44
	45–54
	55–64
	65–74
	Over 75
**Q2. Gender**	Female
	Male
**Q3. Region of Residency**	Open Answer
**Q4. Marital Status**	Unmarried
	Married
	Separated
	Divorced
	Widowed
**Q5. Including yourself, how many people are in your household?**	1
	2
	3
	4
	4+
**Q6. What is your educational level?**	Primary School
	Middle School
	High School
	Bachelor's degree
	Master's degree
	PhD/Specialization School
**Q7. What is your current employment status?**	Employed in a public company
	Employed in a private company
	Self Employed/Freelance
	Entrepreneur

	Housekeeper
	Unemployed
	Student
	Retired
**Q8. Did the coronavirus pandemic reduce the income of your household?**	No, income remained unchanged.
	Yes, income was reduced up to 20%
	Yes, income was reduced between 20 and 50%
	Yes, income was reduced over 50%
	Yes, household income reduced to zero
**Q9. How much do you fear being infected by SARS-CoV-2?**	1 (Not at all)
	2 (A Little)
	3 (Quite)
	4 (A Lot)
	5 (Extremely)
**Q10. How much do you fear SARS-CoV-2 could infect your dearest ones?**	1 (Not at all)
	2 (A Little)
	3 (Quite)
	4 (A Lot)
	5 (Extremely)
**Q11. How much do you feel comfortable when you go out of your home?**	1 (Not at all)
	2 (A Little)
	3 (Quite)
	4 (A Lot)
	5 (Extremely)
**Q12. With respect to the modalities of contagion from coronavirus…**	I feel anxious
	I feel quiet, it is enough to respect the safety measures
	I think the virus has lost its effect, it is less aggressive and contagious than before
**Q13. How much do you think that fear of being infected might generate or has generated collective hysteria?**	1 (Not at all)
	2 (A Little)
	3 (Quite)
	4 (A Lot)
	5 (Extremely)
	Yes

**Q14. Beyond the information received from TVs and newspapers, did you look for more information on COVID-19?**	No
**Q15. Through which channels did you become informed on COVID-19? You can select more than one option.**	Newspapers/Online newspapers
	TV/Radio
	Social Media (Facebook, Instagram, YouTube…)
	Blog/Forum
	Journals or Websites of Medicine, Health, Wellness
	Family Doctor/Other Doctors/Chemist
	Other

Q16. How much do you think these channels are reliable to obtain information on COVID-19? Assign a value from 1 to 5, where 1 is "Not at all" and 5 is "Extremely".

	1	2	3	4	5
Newspapers/Online newspapers					
TV/Radio					
Social Media (Facebook, Instagram, YouTube, etc.)					
Blog/Forum					
Journals or Websites of Medicine, Health, Wellness					
Family Doctor/Other Doctors/Chemist					
Friends and Relatives					

**Q17. How much do you think you are informed about the modalities of contagion from SARS-CoV-2?**	1 (Not at all)
	2 (A Little)
	3 (Quite)
	4 (A Lot)
	5 (Extremely)
	1 (Not at all)
	2 (A Little)

**Q18. How much do you fear a new diffusion of coronavirus after the reopening of activities?**	3 (Quite)
	4 (A Lot)
	5 (Extremely)
**Q19. How much do you think the measures adopted to limit contagion are effective?**	1 (Not at all)
	2 (A Little)
	3 (Quite)
	4 (A Lot)
	5 (Extremely)
**Q20. What is the safety distance between two people who are NOT wearing a face mask?**	At least 1 meter
	2 meters
	More than 2 meters
**Q21. What is the safety distance between two people who are both wearing a face mask?**	At least 1 meter
	2 meters
	More than 2 meters
	The safety distance is not needed
**Q22. If two persons are physically close and just one of them is wearing a face mask, is there a risk of infection?**	Yes
	No
	It depends on the kind of face mask, some of them protect from contagion and at the same time do not allow to infect others
	I do not know
**Q23. During Phase 2, do you think you will resume the activities you left behind at the beginning of the emergency?**	As before the crisis, with no fear
	As before the crisis, with fear
	Less than before, with no fear
	Less than before and with fear
	With fear
	I will not resume
**Q24. What is the main reason for your visits to the dentist?**	I only go for emergencies
	I go for control visits or regular oral hygiene appointments
	I am following a treatment plan
	I do not go / I've never been to a dentist → if so, you can skip directly to question 30
	Yes

**Q25. Do you have a trusted dentist?**	No
**Q26. Which type of dentist clinic are you used to visiting?**	Private
	Public
	Low-Cost Chain
	Affiliated Private Clinic
**Q27. Where is the clinic you visit?**	In the same province and city I live in
	In the same province I live in but in a different city
	In a different province but in the same region I live in
	In a different region from the one I live in
	Abroad
**Q28. For how many years have you visited the same dentist?**	Less than 1 year
	2-3 years
	More than 3 years
**Q29. How often do you go to the dentist?**	In case of necessity
	At least once a year
	A couple of times a year
	Three times or more per year
**Q30. From 9 March to 4 May 2020, did you need to undergo oral care?**	Yes
	No→ if so, you can skip directly to question 34
**Q31. What was your behavior?**	I underwent without any problem
	I underwent with fear
	I postponed them to a date to be determined
	The problem was solved telematically
	The clinic postponed or canceled the appointment
**Q32. How much did these measures make you feel safe? You can choose more than one option.**	The Dentist was wearing Personal Protective Equipment
	I could smell the disinfectant
	I was informed about the precautionary measures adopted
	I looked for information on various channels
	I fully trust my dentist
	I was not feeling safe
	Positively

**Q33. Having undergone the oral care treatment, how do you evaluate the experience?**	Negatively
	I do not know
	Other
**Q34. Do you have any scheduled appointments in the next months?**	Yes
	No
**Q35. How will or would you behave if you have scheduled appointments at the dentist?**	I would undergo oral care without any problem
	I would undergo oral care with fear
	I would postpone the appointment
	I would cancel the appointment
**Q36. What do you think might be the main reason to cancel a scheduled oral care appointment?**	Fear of contagion
	Non-urgent cures
	Lowered income
	I would rather the number of new cases decreases more
	Other
**Q37. During Phase 2, how comfortable would you feel in starting/going on with oral care?**	1 (Not at all)
	2 (A Little)
	3 (Quite)
	4 (A Lot)
	5 (Extremely)
**Q38. During Phase 2, how much do you think the risk of contagion could affect your next appointments?**	1 (Not at all)
	2 (A Little)
	3 (Quite)
	4 (A Lot)
	5 (Extremely)
**Q39. How urgent do you think it is to resume with the cures you stopped?**	1 (Not at all)
	2 (A Little)
	3 (Quite)
	4 (A Lot)
	5 (Extremely)
**Q40. How much do you trust your dentist regarding sterilization and**	1 (Not at all)
	2 (A Little)
	3 (Quite)

**sanitization of tools and environment?**	4 (A Lot)
	5 (Extremely)
**Q41. Did your dentist give you all the information on the sanitization procedures adopted in the dental office?**	Yes
	No
**Q42. Through which means of communication did you obtain the information? You can select more than one option.**	Phone Call
	Talk
	Message
	Email
	Social Network
	I did not receive any information
	Other
**Q43. Will you look for further information to feel safer with concern to access to oral care?**	No, I feel safe and I will not look for other information
	Yes, I will directly call the Clinic or the dentist
	Yes, I will browse the website of the Clinic
	Yes, I will browse websites specialized in medicine
	Yes, I will ask for information on social media
	Yes, I will get informed through friends and family
	Other
